# Cabotegravir + Rilpivirine Long-Acting: Overview of Injection Guidance, Injection Site Reactions, and Best Practices for Intramuscular Injection Administration

**DOI:** 10.1093/ofid/ofae282

**Published:** 2024-05-25

**Authors:** Paula Teichner, Nadine Chamay, Emilie Elliot, Miguel Pascual-Bernáldez, Deanna Merrill, Cindy Garris, Ronald D’Amico, Cecy Felizarta, Emma Torres, Rodica Van Solingen-Ristea, Bryan Baugh, Parul Patel, Vani Vannappagari, Samia Dakhia, Joseph W Polli, Louise Garside, Richard Grove, Shanker Thiagarajah, Eileen Birmingham, Jean van Wyk

**Affiliations:** ViiV Healthcare, Durham, North Carolina, USA; ViiV Healthcare, Brentford, United Kingdom; ViiV Healthcare, Madrid, Spain; ViiV Healthcare, Madrid, Spain; ViiV Healthcare, Durham, North Carolina, USA; ViiV Healthcare, Durham, North Carolina, USA; ViiV Healthcare, Durham, North Carolina, USA; Private practice of Franco Felizarta, MD, Bakersfield, California, USA; Service de maladies infectieuses et tropicales, Hôpital Saint-Antoine, Assistance Publique–Hôpitaux de Paris, Paris, France; Janssen Research and Development, Beerse, Belgium; Janssen Research and Development, Titusville, New Jersey, USA; ViiV Healthcare, Durham, North Carolina, USA; ViiV Healthcare, Durham, North Carolina, USA; ViiV Healthcare, Brentford, United Kingdom; ViiV Healthcare, Durham, North Carolina, USA; PHASTAR, Macclesfield, United Kingdom; GSK, London, United Kingdom; GSK, London, United Kingdom; Janssen Research and Development, Raritan, New Jersey, USA; ViiV Healthcare, Brentford, United Kingdom

**Keywords:** cabotegravir, HIV-1, injection site reaction, long-acting therapy, rilpivirine

## Abstract

**Background:**

Cabotegravir (CAB) + rilpivirine (RPV) dosed monthly or every 2 months is a complete long-acting (LA) regimen for the maintenance of human immunodeficiency virus type 1 virologic suppression. Across the phase 3/3b trials, the most frequently reported adverse events were injection site reactions (ISRs).

**Methods:**

We present pooled ISR characteristics and outcomes for participants receiving CAB + RPV LA through week 96 of the FLAIR and ATLAS-2M studies, and survey results from healthcare providers (HCPs) giving injections (eg, injectors) in the ATLAS, FLAIR, and ATLAS-2M studies to determine optimal injection techniques. Surveys were anonymous, self-administered online questionnaires that queried provider demographics, injection experience, and techniques to minimize pre-/postinjection discomfort. Data were summarized using descriptive statistics.

**Results:**

Overall, 8453 ISRs were reported by 801 participants receiving ≥1 injection of CAB LA/RPV LA. Most ISRs were mild to moderate in severity (grade 1–2, 99%), with a median duration of 3 days (interquartile range, 2–4 days), and rarely led to withdrawal (2%). Surveys were completed by 181 HCPs across 113 sites. Pushing the intramuscular injection at slow speed (66%), bringing the medication to room temperature (58%), and relaxing the gluteus muscle before injecting (53%) were ranked as effective preinjection/injection procedure practices for minimizing pain. Most injectors (60%) indicated that a prone position provided optimal patient comfort, and 41% had no preference on injection medication order.

**Conclusions:**

Taken together, the data demonstrate favorable tolerability with CAB + RPV LA injections over the long term and simple techniques routinely used by injectors to help optimize the administration of CAB + RPV LA injections.

## Teaser

Most injection site reactions were mild to moderate in severity and short-lived, decreased in frequency over time, and infrequently led to withdrawal, demonstrating favorable tolerability with cabotegravir + rilpivirine long-acting injections.

## Graphical Abstract

**Graphical Abstract:**
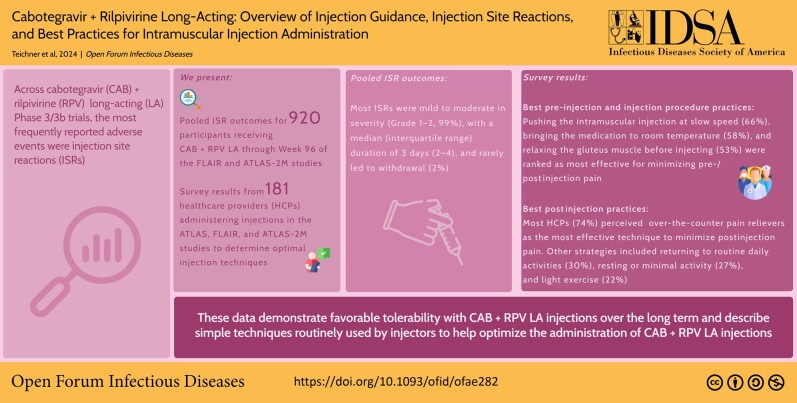
This graphical abstract is also available at Tidbit: https://tidbitapp.io/institutional-portal/open-forum-infectious-diseases/tidbits/cabotegravir-rilpivirine-long-acting-overview-of-injection-guidance-injection-site-reactions-and-best-practices-for-intramuscular-injection-administration

Intramuscular (IM) injections are one of the most frequent clinical procedures performed, with >16 billion administered per year globally [1]. Advantages of the IM route include improved drug absorption and bioavailability and the potential for longer dosing intervals compared with oral therapy [2]. Current medications administered via IM gluteal injections include antibiotics, antipsychotics, hormonal therapies, immunosuppressants, and vaccines [3, 4]. There are 5 potential IM injection sites: deltoid (commonly used for adult vaccinations), dorsogluteal (upper outer quadrant), ventrogluteal (side of the hip), and rectus femoris and vastus lateralis (thigh) [5]. Selecting the optimal location for gluteal injection is important to ensure the safety and success of the injection and to minimize pain [6]. Although preference for the dorsogluteal site has been reported, the ventrogluteal region is safer for IM injection as the site is free of major nerves and blood vessels, with subcutaneous adipose tissue thin enough to reach target muscles [5, 7–9].

Antiretroviral therapy (ART) has continued to evolve, with the development of single-tablet regimens and new drug classes that are better tolerated than previous therapies [10]. However, several challenges associated with daily oral ART remain, including human immunodeficiency virus (HIV) stigma and fear of inadvertent disclosure, anxiety related to adherence, and the daily reminder of HIV status [11]. When people with HIV were asked about ideal treatments, being able to take medicines less frequently was identified as an optimization strategy [12].

Cabotegravir (CAB), an integrase strand transfer inhibitor, plus rilpivirine (RPV), a nonnucleoside reverse transcriptase inhibitor, administered monthly or every 2 months (Q2M) via IM injection is the first complete long-acting (LA) regimen recommended by treatment guidelines for the maintenance of HIV type 1 (HIV-1) virologic suppression [13–15]. Approval of monthly dosing was based on the phase 3 antiretroviral therapy as long-acting supression (ATLAS; NCT02951052) and First Long-Acting Injectable Regimen (FLAIR; NCT02938520) studies, which demonstrated noninferior efficacy of CAB + RPV LA dosed every 4 weeks (Q4W) versus daily oral regimens [16, 17]. Q2M dosing was approved based on the phase 3b antiretroviral Therapy as Long-Acting Supression every 2 Months (ATLAS-2M; NCT03299049) study, which demonstrated noninferiority of CAB + RPV LA dosed every 8 weeks (Q8W) versus Q4W dosing [18, 19]. The longer dosing intervals of CAB + RPV LA may address some of the aforementioned challenges associated with daily oral ART for people with HIV [20, 21]. Additionally, early data suggest that CAB + RPV LA injections can improve adherence compared with daily oral ARTs [22].

CAB + RPV LA represents a new treatment paradigm, as the first LA regimen for the treatment of HIV-1, and an alternative to daily oral ART regimens in virologically suppressed adults and adolescents ≥12 years of age [23]. While some healthcare providers (HCPs) treating HIV may have experience with IM injections for other conditions, such as sexually transmitted infections, some may have little to no experience with this treatment delivery method [24]. Furthermore, although CAB + RPV LA is now implemented in clinical practice globally, there is a lack of evidence-based guidelines for IM injections [5]. Per the prescribing information, CAB LA and RPV LA should be administered at separate IM gluteal injection sites, either on opposite sides or ≥2 cm apart, during the same visit [25]. The ventrogluteal site is recommended, although a dorsogluteal approach is acceptable if preferred by the HCP [25]. Additional recommendations include allowing the medicines to reach room temperature before administration and using longer needle lengths (≥2 inches) for patients with a body mass index (BMI) ≥30 kg/m^2^ to ensure injections are administered intramuscularly instead of subcutaneously [25].

Injection site reactions (ISRs) were the most commonly reported adverse events (AEs) across the CAB + RPV LA phase 2b/3/3b trials [16–19, 21, 26, 27]. The most frequent type of ISR reported following CAB + RPV LA injection was injection site pain, as commonly reported following other IM injections, followed by nodule and induration, which were uncommon [2, 5, 17–19, 21, 26, 27]. While ISRs are frequent in the CAB + RPV LA trials, most were mild to moderate in severity and infrequently led to treatment discontinuation [16–19, 26, 27]. Across the CAB + RPV LA development program, CAB + RPV LA was consistently preferred over daily oral therapy despite the occurrence of ISRs, with 90% of switch participants preferring CAB + RPV LA to once-daily oral bictegravir, emtricitabine, and tenofovir alafenamide (BIC/FTC/TAF) in the phase 3b Switch Onto Long-Acting Therapy (SOLAR) study [20, 21, 28–30]. Injection site pain can reduce patients’ acceptance of treatment and discourage clinicians from using IM injections out of concern for their patients’ comfort [2]. Generally with IM injections, physical and procedural interventions, through the use of an optimal injection technique, have the potential to reduce pain [2, 5]. Examples include allowing medication to reach room temperature, applying manual pressure, and using the ventrogluteal site [2, 5]; however, there is limited published experience of optimal administration of IM injections. It is therefore important to identify techniques to minimize ISRs and optimize CAB + RPV LA IM injection administration to better inform patients/providers.

A wealth of ISR data were collected during the CAB + RPV LA development program. Here, we present long-term pooled ISR outcomes through week 96 from a post hoc analysis of the FLAIR and ATLAS-2M studies. Survey results from injectors who participated in the ATLAS, FLAIR, and ATLAS-2M studies are included to highlight optimal gluteal IM injection techniques and summarize learnings and best practices around injections.

## METHODS

### Study Design and Participants

For the summary of ISR analysis, week 96 data from participants randomized to receive CAB + RPV LA dosed Q8W or Q4W participating in the FLAIR and ATLAS-2M phase 3/3b studies were pooled (Supplementary Figure 1). As the phase 3b SOLAR study had not completed its readout at the time this analysis was conducted, coupled with the fact that SOLAR was also 12 months in duration (<96 weeks), data from this study were not included. The full inclusion/exclusion criteria and study designs have been previously published [16, 19]. In brief, participants were aged ≥18 years and virologically suppressed (plasma HIV-1 RNA <50 copies/mL). FLAIR participants were ART naive at study entry and underwent a 20-week induction phase with a dolutegravir-based 3-drug regimen to achieve virologic suppression. ATLAS-2M participants were ART experienced before entering the study. Most participants entering ATLAS-2M were directly enrolled from either the CAB + RPV LA Q4W or the daily oral comparator arm of the phase 3 ATLAS study. ATLAS-2M participants who had transitioned from ATLAS with prior exposure to CAB + RPV LA were excluded to align duration of exposure. ATLAS data were not included as most participants had transitioned to ATLAS-2M after week 48, before reaching week 96 [18, 19].

A post hoc descriptive injection survey was conducted to explore the injection techniques used to minimize pain and discomfort. Surveys were sent electronically in June 2021 with a 3-month completion period. HCPs who administered injections completed surveys once participants had received at least 96 weeks of therapy in ATLAS, FLAIR, or ATLAS-2M.

### Patient Consent Statement

All 3 studies were conducted in accordance with the Declaration of Helsinki [31]. All participants provided written informed consent, and the study protocols, amendments, informed consent, and other information that required preapproval were reviewed and approved by a national, regional, or investigational center ethics committee or institutional review board.

### Procedures

#### ISR Summary

Pooled ISR data from FLAIR and ATLAS-2M were evaluated by dosing regimen, drug, sex at birth, baseline BMI category, and race (Table 1).

**Table 1.** Baseline Patient Characteristics

Parameter	Pooled CAB + RPV LA (n = 937)	CAB + RPV LA Q8W (n = 327)	CAB + RPV LA Q4W (n = 610)
Participants receiving ≥1 injection	920 (98)	321 (98)	599 (98)
Median age, y (range)	39 (19–83)	42 (20–83)	38 (19–68)
Age, y			
<50	715 (78)	234 (73)	481 (80)
50–64	188 (20)	75 (23)	113 (19)
≥65	17 (2)	12 (4)	5 (<1)
Female (sex at birth)	201 (22)	70 (22)	131 (22)
Race			
White	698 (76)	234 (73)	464 (77)
Black	147 (16)	57 (18)	90 (15)
Asian	39 (4)	16 (5)	23 (4)
Other^a^	36 (4)	14 (4)	22 (4)
Median BMI, kg/m^2^ (IQR)	24.9 (22.6–28.0)	25.3 (22.8–28.6)	24.8 (22.5–27.8)
BMI <30 kg/m^2^	770 (84)	262 (82)	508 (85)
BMI ≥30 kg/m^2^	150 (16)	59 (18)	91 (15)

Data are presented as No. (%) unless otherwise indicated.

Abbreviations: BMI, body mass index; CAB, cabotegravir; IQR, interquartile range; LA, long-acting; Q4W, every 4 weeks; Q8W, every 8 weeks; RPV, rilpivirine; y, years.

^a^“Other” includes participants who were American Indian or Alaska Native (64% [n = 23/36]), Native Hawaiian or other Pacific Islander (11% [n = 4/36]), or of multiple races (25% [n = 9/36]).

ISR data were proactively collected by HCPs, who identified and reported any new or resolving ISRs before and after CAB + RPV LA administration, from a preidentified list or as free text. Each ISR was counted separately; a participant may have had multiple ISR events following a single injection. ISR characteristics, including type, duration, and severity, were summarized descriptively using the Division of AIDS grading system (grades 1–5).

#### Injection Survey

HCPs who administered injections in the ATLAS, FLAIR, and ATLAS-2M studies completed an anonymous, voluntary, and self-administered online survey. The questionnaire, sent to 150 sites across 15 countries, contained 15 items with predefined response options and 1 open-ended item (Supplementary material: Questionnaire). Study sites were asked to offer the survey to any HCPs who administered CAB + RPV LA injections during the study period; therefore, the total number of HCPs who were sent the survey is not available. Topics included provider demographics, prior clinical and injection experience, techniques to minimize pre- and postinjection discomfort, and perceived effectiveness of these techniques (ranking based on the number of HCPs who reported ≥1 technique as effective). These data were summarized using descriptive statistics.

## RESULTS

### ISR Summary

A total of 937 (FLAIR, n = 283; ATLAS-2M, n = 654) participants naive to CAB + RPV LA were randomized and 920 received ≥1 dose of CAB + RPV LA (FLAIR, Q4W only: n = 283; ATLAS-2M: Q8W, n = 327 and Q4W, n = 327). The median age was 39 years, 22% were female (sex at birth), 15% were Black, and the median BMI was 24.9 kg/m^2^. Baseline characteristics were broadly similar between treatment groups (Table 1).

Among 34 939 CAB + RPV LA injections administered in the FLAIR and ATLAS-2M trials, 8453 ISR events were reported through week 96. The most commonly reported ISR event (as a percentage of injections received) was injection site pain (20%); nodule (1%), induration (<1%), discomfort (<1%), and swelling (<1%) were reported infrequently (Table 2). Injection site necrosis, injection site fibrosis, and injection site scars were rarely reported (all <1%) and led to the withdrawal of 1 participant (injection site necrosis). The frequency of ISRs was generally comparable by drug; however, numerically more injection site pain events were reported with RPV compared with CAB (22% vs 18% of injections).

**Table 2.** Injection Site Reaction Events Through Week 96 by Dosing Regimen and Drug

Parameter	CAB + RPV LA Dosing Regimen			Drug^a^	
	CAB + RPV LA Q8W (n = 327)	CAB + RPV LA Q4W (n = 610)	Total (n = 937)	CAB (n = 937)	RPV (n = 937)
No. (%) of participants receiving ≥1 injection	321 (98)	599 (98)	920 (98)	920 (98)	920 (98)
No. of injections	7954	26 985	34 939	17 468	17 471
No. of ISR events (event-level)^b^	2345	6108	8453	3836	4606
Pain, No. (% of injections)	1904 (24)	5035 (19)	6939 (20)	3142 (18)	3789 (22)
Nodule, No. (% of injections)^c^	107 (1)	355 (1)	462 (1)	213 (1)	249 (1)
Induration, No. (% of injections)	61 (<1)	208 (<1)	269 (<1)	130 (<1)	139 (<1)
Discomfort, No. (% of injections)	113 (1)	107 (<1)	220 (<1)	112 (<1)	107 (<1)
Swelling, No. (% of injections)	56 (<1)	85 (<1)	141 (<1)	65 (<1)	76 (<1)
Grade, No. (%)					
Grade 1	1843 (79)	5157 (84)	7000 (83)	3112 (81)	3880 (84)
Grade 2	468 (20)	889 (15)	1357 (16)	678 (18)	676 (15)
Grade 3^d^	34 (1)	62 (1)	96 (1)	46 (1)	50 (1)
Participants withdrawing due to injection-related reasons, No. (% of participants with ≥1 injection)^e^	5 (2)	15 (3)	20 (2)	7 (<1)^f^	9 (<1)^f^

Abbreviations: CAB, cabotegravir; ISR, injection site reaction; LA, long-acting; Q4W, every 4 weeks; Q8W, every 8 weeks; RPV, rilpivirine.

^a^As per the trial protocols, HCPs were advised to administer CAB and RPV injections on different sides of the body (eg, left and right gluteus medius) or spaced approximately 2 cm from one another, from the site of any previous injection, or from any previous ISRs. The time, side, and location of CAB and RPV injections were reported.

^b^Each ISR event was counted separately. A participant may have had multiple ISR events following a single injection. The top 5 most common ISRs are reported. Less common ISR events reported included (event-level) pruritus (n = 131), warmth (n = 81), erythema (n = 67), bruising (n = 43), anesthesia (n = 21), hematoma (n = 21), reaction (n = 18), discoloration (n = 10), hemorrhage (n = 5), abscess (n = 4), rash (n = 3), necrosis (n = 3), fibrosis (n = 2), discharge (n = 2), papule (n = 2), cyst (n = 2), movement impairment (n = 2), scar (n = 2), mass (n = 1), hypoesthesia (n = 1), granuloma (n = 1).

^c^Four hundred sixty-two injection site nodule events were reported by 19% (n = 178/920) of all participants who received ≥1 injection; of these events, 80% (n = 373/462) occurred in the same 89 participants.

^d^There were no grade 4 or 5 ISR events. The few grade 3 ISRs included discomfort (3%), pain (1%), induration (<1%), and swelling (<1%).

^e^Owing to 1 or more ISR events (n = 10) or injection intolerability (n = 10).

^f^Excludes participants withdrawing due to injection intolerability. Participants withdrawing due to ISR adverse events by drug were not mutually exclusive.

Most ISRs were mild to moderate in severity (grade 1, 83%; grade 2, 16%); the few grade 3 ISRs (as a percentage of ISR events) included discomfort (3%), pain (1%), induration (<1%), and swelling (<1%). No grade 4 or 5 ISR events were reported through week 96. Withdrawals due to injection-related reasons occurred in 2% of participants and were comparable between drug and dosing regimens but differed by race, with a larger proportion of Asian participants withdrawing for injection-related reasons (10% of participants with injections) versus other races (White, 2%; Black, <1%; Other races, 0% [see Table 1 for breakdown of Other races]).

The median duration of ISRs was 3 days (interquartile range, 2–4), with 87% of ISR events lasting ≤7 days, and no differences observed by drug or dosing regimen (Supplementary Table 1). Overall, 99% of ISRs were self-limited, with approximately 1% of events reported as still resolving or not recovered at the time of data analysis. Of the 8453 ISR events, 28 were reported as “not recovered/resolved” and consisted of pain (32% [n = 9/28]), pruritus (25% [n = 7/28]), nodule (21% [n = 6/28]), discomfort (7% [n = 2/28]), fibrosis (7% [n = 2/28]), induration (4% [n = 1/28]), and necrosis (4% [n = 1/28]). Some ISR events were reported as “recovered with sequelae” (n = 59), comprising pain (69% [n = 41/59]), induration (10% [n = 6/59]), nodule (5% [n = 3/59]), swelling (5% [n = 3/59]), and discomfort (2% [n = 1/59]). Among injection site pain and discomfort events, 91%–93% resolved in ≤7 days (Supplementary Figure 2). Among injection site swelling, induration, and nodule events, 67%, 46%, and 41% recovered in ≤7 days, respectively. Injection site nodule and injection site induration events were reported to have taken the longest to resolve, with a median time to resolution of 9 and 8 days, respectively; most were grade 1 or 2 in severity (injection site nodule: grade 1, 86% [n = 398/462]; grade 2, 14% [n = 64/462]; injection site induration: grade 1, 91% [n = 245/269]; grade 2, 9% [n = 23/269]), with a single grade 3 event of injection site induration reported (duration 3 days, self-resolving). Of the 462 injection site nodule events reported by 19% of all participants, 80% (n = 373/462) occurred in the same 89 participants. The proportion of injection site nodule events reported were similar by baseline BMI category and drug; however, nodule events were reported by a slightly higher proportion of female versus male (sex at birth) participants (24% [n = 48/201] vs 18% [n = 130/719], respectively), participants in the Other races category (44% [n = 16/36]) versus Black (22% [n = 32/147]), White (18% [n = 124/698]), and Asian (13% [n = 5/39]) categories, as well as numerically more participants receiving Q4W versus Q8W dosing (23% [n =137/610] vs 13% [n = 41/321], respectively).

ISRs decreased in incidence over time, reported by 71%, 24%, and 15% of participants at week 4, week 48, and week 96, respectively, and were comparable between dosing regimens (Supplementary Figure 3). When examining ISR profiles across subgroups (sex, race, and BMI category), the frequency, type, and severity of ISRs reported were generally similar, except for slightly numerically fewer injection site pain events reported in female (sex at birth) participants (17% vs 21% in male participants), participants with BMI ≥30 kg/m^2^ (16% vs 21% in participants with BMI <30 kg/m^2^), and Black participants (13% vs 21% in White participants) (Table 3).

**Table 3.** Injection Site Reaction Events Through Week 96 by Demographics

Parameter	Sex at Birth		Baseline BMI Category		Race			
	Female (n = 211)	Male (n = 726)	<30 kg/m^2^ (n = 786)	≥30 kg/m^2^ (n = 151)	White (n = 711)	Black (n = 149)	Asian (n = 41)	Other^a^ (n = 36)
No. (%) of participants receiving ≥1 injection	201 (95)	719 (99)	770 (98)	150 (99)	698 (98)	147 (99)	39 (95)	36 (100)
No. of injections	7617	27 322	29 428	5511	26 587	5630	1360	1362
No. of ISR events (event-level)^b^	1840	6613	7304	1149	6655	911	435	452
Pain, No. (% of injections)	1322 (17)	5617 (21)	6062 (21)	877 (16)	5520 (21)	705 (13)	378 (28)	336 (25)
Nodule, No. (% of injections)	121 (2)	341 (1)	381 (1)	81 (1)	286 (1)	88 (2)	30 (2)	58 (4)^c^
Induration, No. (% of injections)	156 (2)	113 (<1)	235 (<1)	34 (<1)	246 (<1)	11 (<1)	4 (<1)	8 (<1)
Discomfort, No. (% of injections)	18 (<1)	202 (<1)	162 (<1)	58 (1)	192 (<1)	18 (<1)	5 (<1)	5 (<1)
Swelling, No. (% of injections)	45 (<1)	96 (<1)	119 (<1)	22 (<1)	94 (<1)	36 (1)	2 (<1)	9 (<1)
Grade								
Grade 1, No. (% of ISR events)	1585 (86)	5415 (82)	6090 (83)	910 (79)	5567 (84)	708 (78)	373 (86)	352 (78)
Grade 2, No. (% of ISR events)	246 (13)	1111 (17)	1151 (16)	206 (18)	1007 (15)	194 (21)	62 (14)	94 (21)
Grade 3, No. (% of ISR events)^d^	9 (<1)	87 (1)	63 (<1)	33 (3)	81 (1)	9 (<1)	0	6 (1)
Median duration, d (IQR)	3 (2–7)	3 (2–4)	3 (2–4)	3 (2–5)	3 (2–4)	4 (2–7)	2 (1–3)	3 (2–4)
Participants withdrawing due to injection-related reasons, No. (% of participants with ≥1 injection)	2 (<1)	18 (2)	19 (2)	1 (<1)	15 (2)	1 (<1)	4 (10)^e^	0

Abbreviations: BMI, body mass index; IQR, interquartile range; ISR, injection site reaction.

^a^“Other” includes participants who were American Indian or Alaska Native (64% [n = 23/36]), Native Hawaiian or other Pacific Islander (11% [n = 4/36]), or of multiple races (25% [n = 9/36]).

^b^Top 5 most common ISRs overall reported. Each ISR event was counted separately. A participant may have had multiple ISR events following a single injection.

^c^The proportion of participants reporting injection site nodule events was higher in participants of Other races compared with Black, White, and Asian participants; however, the number of participants of Other races in the analysis was low (n = 36/937).

^d^There were no grade 4 or 5 ISR events.

^e^The rate of withdrawals due to injection-related reasons was numerically higher in Asian participants than in White, Black, or Other race categories; however, the number of Asian participants in the analysis was low (n = 41/937).

### Injection Survey

Overall, 181 HCPs returned the survey, most of whom were licensed nurses or medical doctors (Table 4, Supplementary Figure 4). Survey responses were received from 75% (n = 113/150) of sites that participated in ATLAS, FLAIR, and ATLAS-2M, with a mean response of 1.6 HCPs per site (range, 1–8) among responding sites. Overall, 46% of HCPs reported having >10 years of prior experience administering gluteal injections before CAB + RPV LA study participation. During the study, 57% of HCPs administered CAB + RPV LA to ≤10 participants, and 56% delivered ≥100 CAB + RPV LA injections. These results were largely consistent across regions.

**Table 4.** Baseline Healthcare Provider Characteristics

Parameter	Total (n = 181)	North America^a^ (n = 61)	Europe^b^ (n = 71)	Outside of the US, Canada, and Europe^c^ (n = 49)
Role of HCP				
Medical doctor	44 (24)	4 (7)	10 (14)	30 (61)
Licensed nurse	99 (55)	32 (52)	57 (80)	10 (20)
NP/prescribing nurse	14 (8)	4 (7)	3 (4)	7 (14)
Physician assistant	2 (1)	2 (3)	0	0
Medical assistant^d^	9 (5)	9 (15)	0	0
Pharmacist	1 (<1)	1 (2)	0	0
Other	12 (7)	9 (15)	1 (1)	2 (4)
No. of years administering gluteal injections				
0–5	76 (42)	30 (49)	20 (28)	26 (53)
6–10	22 (12)	8 (13)	8 (11)	6 (12)
11–20	29 (16)	13 (21)	12 (17)	4 (8)
>20	54 (30)	10 (16)	31 (44)	13 (27)
No. of participants the HCP injected with CAB + RPV LA				
≤10	103 (57)	30 (49)	42 (59)	31 (63)
11–25	52 (29)	23 (38)	17 (24)	12 (24)
26–50	8 (4)	4 (7)	3 (4)	1 (2)
>50	18 (10)	4 (7)	9 (13)	5 (10)
No. of injections of CAB and RPV administered by the HCP^e^				
6–19	20 (11)	6 (10)	5 (7)	9 (18)
20–49	23 (13)	6 (10)	12 (17)	5 (10)
50–99	36 (20)	12 (20)	16 (23)	8 (16)
≥100	102 (56)	37 (61)	38 (54)	27 (55)

Data are presented as No. (%) unless otherwise indicated.

Abbreviations: CAB, cabotegravir; HCP, healthcare provider; LA, long-acting; NP, nurse practitioner; RPV, rilpivirine; US, United States.

^a^US (25% [n = 45]), Canada (9% [n = 16]).

^b^France (12% [n = 22]), Italy (1% [n = 2]), Spain (20% [n = 37]), Sweden (2% [n = 3]), the Netherlands (1% [n = 2]), and the United Kingdom (3% [n = 5]).

^c^Argentina (16% [n = 8]), Australia (10% [n = 5]), Japan (16% [n = 8]), Mexico (16% [n = 8]), Republic of Korea/South Korea (8% [n = 4]), Republic of South Africa (10% [n = 5]), and Russia (22% [n = 11]).

^d^Healthcare assistant, United Kingdom.

^e^CAB and RPV administered during the same visit were counted as separate injections.

**Figure 1.**
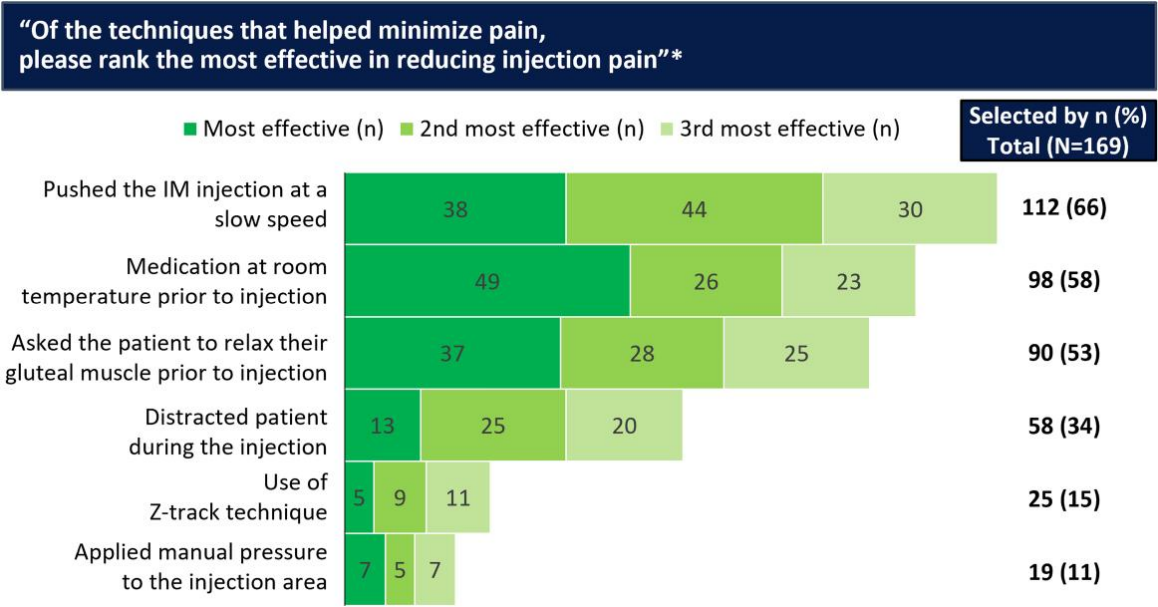
Pain- and discomfort-minimizing techniques prior to or during injection. *Those reported by ≥10% of healthcare providers are shown. Those ranked by <10% included use of ventrogluteal site for injection (7%), administered pain relief prior to injection (7%), use of dorsogluteal site for injection (6%), used a smaller-bore needle (ie, 25 gauge) for the injection (5%), use of a different needle length (other than 1.5 inches) to accommodate body type (5%), use of a topical or injectable anesthetic (ie, lidocaine; 2%), pushed the intramuscular (IM) injection at a fast speed (1%), applied a hot pack just prior to injection (1%), applied a cold pack just prior to injection (0%), and “other” techniques (4%).

Among HCPs who reported using ≥1 injection technique, pushing the IM injection at a slow speed (determination of “slow” was at the discretion of the HCP; 66%), bringing the medication to room temperature (58%), relaxing the gluteal muscle before injection (53%), and distracting the patient (34%) were ranked as most effective preinjection/injection procedure practices effective for minimizing pain (Figure 1). Across regions, most (60%) injectors also reported that putting the patient into the prone position provided optimal patient comfort (Supplementary Figure 5). When asked their opinion on why patient reporting of pain declines over time, most HCPs perceived this to be due to decreased anxiety (82%) and improvements in patient self-management postinjection (75%); these observations were consistent across regions (Supplementary Figure 6).

One in 4 HCPs modified their injection technique for patients with BMI ≥30 kg/m^2^ (Supplementary Figure 7). This trend was higher in North America (39% [n = 24/61]) and Europe (25% [n = 18/71]) than in regions outside of the United States (US), Canada, and Europe (10% [n = 5/49]); however, the number of HCPs responding to this question differed by region. The most common injection modification was using a longer needle (ie, 2 inches; 98% of HCPs reporting technique modification), followed by using the Z-track technique (23%), positioning the patient differently (4%), and using a different landmarking method to locate the injection site (4%).

There was no consensus on the preference for CAB or RPV to be injected first; however, HCPs in regions outside of the US, Canada, and Europe were more likely to prefer injecting CAB before RPV (primarily HCPs in Argentina, Mexico, and Russia).

When asked how often they used the ventrogluteal or dorsogluteal injection location, HCPs in North America utilized ventrogluteal injections more commonly than dorsogluteal injections (76% vs 24% of injections). In comparison, dorsogluteal injections were more common in Europe (78%) and regions outside of the US, Canada, and Europe (74%), except HCPs who responded from the Netherlands (100%), Sweden (98%), and Australia (82%), who used ventrogluteal injections more frequently; however, few HCPs in these countries responded (n = 2–5 per country).

Most HCPs (74%) perceived over-the-counter pain relievers as the most effective technique to minimize postinjection pain (Supplementary Figure 8). Other strategies included returning to routine daily activities (30%), resting or minimal activity (27%), and light exercise (22%).

## DISCUSSION

This analysis utilizes data collected from diverse participants and HCPs who participated in CAB + RPV LA phase 3/3b trials to characterize the ISR profile and to provide insights on optimal gluteal IM injection technique.

Injection site pain was the most commonly reported ISR, as also observed in the earlier Long-Acting Antiretroviral Treatment Enabling Trial 2 (LATTE-2), Oral to Long Acting Rollover (POLAR), and ATLAS studies, and consistent with LA IM injectable antipsychotic medications for treating schizophrenia [27, 32–37]. Injection site nodule, discomfort, and swelling also occurred across the phase 2b/3/3b program, although far less frequently than pain events [17–19, 26, 27].

The incidence, type, and severity of ISRs reported were generally comparable by dosing regimen, drug, sex at birth, baseline BMI category, and race, with small numerical differences noted. Injection site nodule was reported by a slightly higher proportion of female (sex at birth) participants and participants in the “Other races” category, and numerically more participants receiving Q4W dosing; however, the higher rate of injection site nodule events in participants of Other races versus the other categories may be due to the low number of participants in this subgroup. Injection site pain events were reported less frequently in women, participants of Black race, individuals with a higher BMI (≥30 kg/m^2^), those receiving Q4W dosing, and those receiving CAB injections.

Most ISRs were mild to moderate in severity and short-lived, consistent with the results of the individual study reports [17, 18, 38]. The incidence of ISR events decreased over time through week 96, an observation consistent with parenteral treatment for other conditions [39–41]; most participants recovered fully within 7 days.

Injection-related reasons for withdrawal were infrequent and comparable between dosing regimens, with participant retention rates similar to the daily comparator arm in FLAIR [18, 38]. Conclusions cannot be drawn from the higher rate of withdrawals due to injection-related reasons for Asian participants versus other races, on account of the low number of Asian participants in the analysis. As previously reported, patient-reported outcome measures at week 48 of the ATLAS, FLAIR, and ATLAS-2M trials demonstrated high levels of treatment satisfaction and acceptance for CAB + RPV LA, with most participants rating injections as “totally” or “very acceptable” [28, 29]. Furthermore, most participants preferred CAB + RPV LA versus daily oral ART, with 90% of switch participants preferring CAB + RPV LA to once-daily oral BIC/FTC/TAF in the phase 3b SOLAR study [20, 21, 28–30]. Collectively, the high rates of treatment acceptance, treatment satisfaction, and participant preference for CAB + RPV LA reported across the development program suggest a desire to continue LA treatment for most participants, despite the occurrence of ISRs [28, 29].

Data from the surveys completed by injectors across the development program highlight several techniques to optimize the administration of CAB + RPV LA injections. The most effective techniques perceived by HCPs to minimize pain before/during injections were pushing the injection slowly, bringing the medication to room temperature, and having the patient relax their gluteal muscle before injection, consistent with the Cabotegravir and Rilpivirine Implementation Study in European Locations (CARISEL) implementation–effectiveness study [42]. These simple techniques are straightforward to implement and should be considered when administering CAB + RPV LA to minimize pain during IM injection. Over-the-counter pain relievers and returning to daily activities were perceived by HCPs as the most effective techniques for minimizing postinjection pain; however, it should be noted that, as most pain events were self-limited, over-the-counter pain relievers are unlikely to be required in most cases. HCPs should counsel patients on these techniques and other potential postinjection strategies (resting or minimal activity, light exercise, cold packs, hot packs) to minimize discomfort. These findings were broadly comparable across regions, reinforcing that simple techniques routinely used by injectors can be used to optimize the administration of CAB + RPV LA injections.

Using a longer needle was the most common modification made by HCPs for patients with a higher BMI, aligned with treatment recommendations to accommodate body habitus for patients with a BMI ≥30 kg/m^2^ [43]. It has been shown previously that participants with a higher BMI have a slower rate of absorption from the depot compared with those with a lower BMI [43–45]. The utilization of longer 2-inch needles can help mitigate this, with data showing higher median CAB trough concentrations early in treatment in participants with a BMI ≥30 kg/m^2^ who received injections using a 2-inch needle versus those receiving injections with a <2-inch needle [43]. Therefore, using longer needles in individuals with a high BMI can help reach the muscle and assure the medications are injected appropriately, which is particularly important given the rising prevalence of obesity globally [1]. A post hoc analysis to identify factors associated with an increased risk for confirmed virologic failure (CVF) showed that a BMI ≥30 kg/m^2^, when present in combination with ≥1 additional baseline risk factor (baseline RPV resistance-associated mutations or HIV-1 subtype A6/A1), was associated with an increased risk of CVF [46]. However, participants with BMI ≥30 kg/m^2^ as the only baseline risk factor had a CVF rate of 0.5%, similar to those with no baseline factors [43, 47].

Research is ongoing into alternative modes of injection to extend the dosing interval and improve tolerability and accessibility (eg, in the context of buttock cosmetic procedures); this includes the evaluation of alternative sites of administration (eg, vastus lateralis thigh muscle) and alternative routes of administration (eg, subcutaneous). For instance, in participants at steady state, with ≥3 years of treatment with gluteal IM injections, CAB and RPV pharmacokinetic profiles after 16 weeks of IM thigh injections (4 injections Q4W or 2 injections Q8W) were similar to those following gluteal administration, with no clinically significant differences observed [48]. These data from the ATLAS-2M substudy, alongside population pharmacokinetic simulations [49], support the potential of rotational/short-term CAB + RPV LA IM lateral thigh administration within an established gluteal regimen; however, longer-term efficacy and safety data are needed to better characterize the role of LA thigh administration in the treatment of HIV. It should be noted that this approach has not been approved by regulatory bodies.

The lack of blinding for the CAB + RPV LA administration in the FLAIR and ATLAS-2M studies may have caused participants to anticipate and report more AEs [50]. Safety assessments were performed more frequently for participants in the Q4W arm than in the Q8W arm, which may have increased the number of AEs reported in the Q4W arm. This analysis was not adequately powered to draw any statistical ISR inferences between dosing regimens or other subgroups examined. Furthermore, the small size of some subgroups should be noted. For example, the numbers of Asian participants (n = 41/937) and participants of Other races (n = 36/937) were low.

## CONCLUSIONS

Most ISRs were mild to moderate in severity and short-lived, decreased in frequency over time, and infrequently led to withdrawal, demonstrating favorable tolerability with CAB + RPV LA injections dosed monthly and Q2M over the long term. Data from the survey of HCPs experienced in administering IM injections reinforce that simple techniques routinely used by injectors help optimize the administration and experience of CAB + RPV LA injections.

## Supplementary Data

Supplementary materials are available at *Open Forum Infectious Diseases* online. Consisting of data provided by the authors to benefit the reader, the posted materials are not copyedited and are the sole responsibility of the authors, so questions or comments should be addressed to the corresponding author.

## Supplementary Material

ofae282_Supplementary_Data

## Notes

***Acknowledgments.*** The authors thank everyone who has contributed to the success of the ATLAS, FLAIR, and ATLAS-2M studies, all study participants and their families, and the clinical investigators and their staff. The ATLAS, FLAIR, and ATLAS-2M studies were funded by ViiV Healthcare and Janssen Research and Development; this analysis was funded by ViiV Healthcare. Editorial assistance was provided by Poppie Cooper at Nucleus Global, funded by ViiV Healthcare.

***Author contributions.*** M. P.-B. and D. M. assisted with study implementation and data acquisition. P. T., N. C., E. E., C. G., R. D., R. V. S.-R., B. B., P. P., V. V., S. D., J. W. P., L. G., R. G., S. T., E. B., and J. v. W. participated in the analysis of the study data and the conceptualization and design of the studies. All authors were involved in the drafting and review of the manuscript and approved the final version.

***Disclaimer.*** The funders participated in the collection, analysis, and interpretation of data, in the writing of the report, and in the decision to submit the paper for publication.

***Data availability.*** Data sharing requests will be considered by the management group upon written request to the corresponding author. De-identified participant data or other prespecified data will be available subject to a written proposal and a signed data sharing agreement.

***Financial support*.** This work was supported by ViiV Healthcare and Janssen Research & Development. All authors vouch for the accuracy and completeness of the data, data analyses, and interpretation and fidelity to the protocol, and all have approved the final manuscript for submission.

***Potential conflicts of interest*.** P. T., N. C., E. E., M. P.-B., D. M., C. G., R. D., P. P., V. V., S. D., J. W. P., and J. v. W. are employees of ViiV Healthcare and may be stockholders of GSK. C. F. has received speaking fees from ViiV Healthcare. R. V. S.-R., B. B., and E. B. are employees of and may be stockholders of Janssen, Pharmaceutical Companies of Johnson & Johnson. L. G., R. G., and S. T. are employees, and may be stockholders, of GSK. E. T. reports no potential conflicts.
