# Supplementary material for: Cabotegravir + Rilpivirine Long-Acting: Overview of Injection Guidance, Injection Site Reactions, and Best Practices for Intramuscular Injection Administration
Source: Open Forum Infect Dis. 2024 May 25;11(6):ofae282. doi: 10.1093/ofid/ofae282 (PMC11179104; doi:10.1093/ofid/ofae282)
Supplement: ofae282_Supplementary_Data [file ofae282_supplementary_data.docx]

Supplement

Supplementary Material: Questionnaire

1. **Please select the country where your site is located.**

***Please select one answer.***

| 1 | ⭘ | Argentina |
| --- | --- | --- |
| 2 | ⭘ | Australia |
| 3 | ⭘ | Canada |
| 4 | ⭘ | France |
| 5 | ⭘ | Germany |
| 6 | ⭘ | Italy |
| 7 | ⭘ | Japan |
| 8 | ⭘ | The Netherlands |
| 9 | ⭘ | Republic of Korea |
| 10 | ⭘ | Mexico |
| 11 | ⭘ | Russia |
| 12 | ⭘ | Republic of South Africa |
| 13 | ⭘ | Spain |
| 14 | ⭘ | Sweden |
| 15 | ⭘ | United Kingdom |
| 16 | ⭘ | United States of America |

1. **As a healthcare provider administering CAB + RPV LA injections to study participants at your site, please specify your role:**

***Please select one answer.***

| 1 | ⭘ | Medical doctor |
| --- | --- | --- |
| 2 | ⭘ | Licensed nurse |
| 3 | ⭘ | Nurse practitioner/prescribing nurse |
| 4 | ⭘ | Physician assistant |
| 5 | ⭘ | Medical assistant |
| 6 | ⭘ | Pharmacist |
| 7 | ⭘ | Other |

1. **How many years have you been practicing this role:**

***Please select one answer.***

| 1 | ⭘ | 0–5 years |
| --- | --- | --- |
| 2 | ⭘ | 6–10 years |
| 3 | ⭘ | 11–20 years |
| 4 | ⭘ | >20 years |

1. **How many years’ practice in gluteal injection administration did you have before participating in a CAB + RPV LA trial as an injector:**

***Please select one answer.***

| 1 | ⭘ | 0–5 years |
| --- | --- | --- |
| 2 | ⭘ | 6–10 years |
| 3 | ⭘ | 11–20 years |
| 4 | ⭘ | >20 years |

1. **In which Phase III studies have you participated where you have administered CAB + RPV LA injections?**

***Please select all that apply.***

| 1 | 🞏 | ATLAS |
| --- | --- | --- |
| 2 | 🞏 | FLAIR |
| 3 | 🞏 | ATLAS-2M |

1. **Approximately how many study participants have you personally injected with CAB + RPV LA?**

***Please select one answer.***

| 1 | ⭘ | ≤10 participants |
| --- | --- | --- |
| 2 | ⭘ | 11–25 participants |
| 3 | ⭘ | 26–50 participants |
| 4 | ⭘ | >50 participants |

1. **Approximately how many injections of CAB and RPV have you personally administered? (count CAB and RPV administered during the same visit as separate injections)?**

***Please select one answer.***

| 1 | ⭘ | 1–5 injections **<CLOSE IF SELECTED>** |
| --- | --- | --- |
| 2 | ⭘ | 6–19 injections |
| 3 | ⭘ | 20–49 injections |
| 4 | ⭘ | 50–99 injections |
| 5 | ⭘ | 100+ injections |

Thank you for completing the screening questions; you are invited to participate in this survey.

**8. Which of the following techniques did you utilize to help minimize pain or discomfort of the actual injection prior to or during the injection?**

***Please select all that apply.***

| 1 | 🞏 | Applied manual pressure to the injection area |
| --- | --- | --- |
| 2 | 🞏 | Assured medication was at room temperature prior to administering |
| 3 | 🞏 | Applied hot pack just prior to injection |
| 4 | 🞏 | Applied cold pack just prior to injection |
| 5 | 🞏 | Distracted patient during the injection |
| 6 | 🞏 | Use of ventrogluteal site for the injection |
| 7 | 🞏 | Use of dorsogluteal site for the injection |
| 8 | 🞏 | Use of Z-track technique |
| 9 | 🞏 | Used a smaller bore needle (i.e. 25 gauge) for the injection |
| 10 | 🞏 | Used a different needle length (other than 1.5 inch) to accommodate body type |
| 11 | 🞏 | Pushed the IM injection at a slow speed |
| 12 | 🞏 | Pushed the IM injection at a fast speed |
| 13 | 🞏 | Asked the patient to relax their gluteus muscle prior to injection |
| 14 | 🞏 | Administered a pain reliever prior to injection |
| 15 | 🞏 | Used topical or injectable anesthetic (i.e. lidocaine) |
| 16 | ⭘ | None of these |
| 17 | 🞏 | Other __________ |

**8.1 For each technique choice selected above, please indicate impact of the strategy on pain or discomfort of injection.**

***Please select one answer per row.***

|  |  | 1 | 2 | 3 | 4 |
| --- | --- | --- | --- | --- | --- |
|  |  | **Helped minimize** pain or discomfort of the injection | Made pain or discomfort of the injection **worse** | **No impact** on pain or discomfort of the injection | **Not sure** of the impact on pain or discomfort of the injection |
| 1 | Applied manual pressure to the injection area | ⭘ | ⭘ | ⭘ | ⭘ |
| 2 | Assured medication was at room temperature prior to administering | ⭘ | ⭘ | ⭘ | ⭘ |
| 3 | Applied hot pack just prior to injection | ⭘ | ⭘ | ⭘ | ⭘ |
| 4 | Applied cold pack just prior to injection | ⭘ | ⭘ | ⭘ | ⭘ |
| 5 | Distracted patient during the injection | ⭘ | ⭘ | ⭘ | ⭘ |
| 6 | Use of ventrogluteal site for the injection | ⭘ | ⭘ | ⭘ | ⭘ |
| 7 | Use of dorsogluteal site for the injection | ⭘ | ⭘ | ⭘ | ⭘ |
| 8 | Use of Z-track technique | ⭘ | ⭘ | ⭘ | ⭘ |
| 9 | Used a smaller bore needle (i.e. 25 gauge) for the injection | ⭘ | ⭘ | ⭘ | ⭘ |
| 10 | Used a different needle length (other than 1.5 inch) to accommodate body type | ⭘ | ⭘ | ⭘ | ⭘ |
| 11 | Pushed the IM injection at a slow speed | ⭘ | ⭘ | ⭘ | ⭘ |
| 12 | Pushed the IM injection at a fast speed | ⭘ | ⭘ | ⭘ | ⭘ |
| 13 | Asked the patient to relax their gluteus muscle prior to injection | ⭘ | ⭘ | ⭘ | ⭘ |
| 14 | Administered a pain reliever prior to injection | ⭘ | ⭘ | ⭘ | ⭘ |
| 15 | Used topical or injectable anesthetic (i.e. lidocaine) | ⭘ | ⭘ | ⭘ | ⭘ |
| 17 | Other: **[PIPE THROUGH WORDING FROM Q8 (CODE 17)]** | ⭘ | ⭘ | ⭘ | ⭘ |

**8.2 Of those that helped minimize pain above,** **please rank the most effective in reducing injection pain:**

|  |  | **Most effective** in reducing injection pain | **Second most effective** in reducing injection pain | **Third most effective** in reducing injection pain |
| --- | --- | --- | --- | --- |
| 1 | Applied manual pressure to the injection area | ⭘ | ⭘ | ⭘ |
| 2 | Assured medication was at room temperature prior to administering | ⭘ | ⭘ | ⭘ |
| 3 | Applied hot pack just prior to injection | ⭘ | ⭘ | ⭘ |
| 4 | Applied cold pack just prior to injection | ⭘ | ⭘ | ⭘ |
| 5 | Distracted patient during the injection | ⭘ | ⭘ | ⭘ |
| 6 | Use of ventrogluteal site for the injection | ⭘ | ⭘ | ⭘ |
| 7 | Use of dorsogluteal site for the injection | ⭘ | ⭘ | ⭘ |
| 8 | Use of Z-track technique | ⭘ | ⭘ | ⭘ |
| 9 | Used a smaller bore needle (i.e. 25 gauge) for the injection | ⭘ | ⭘ | ⭘ |
| 10 | Used a different needle length (other than 1.5 inch) to accommodate body type | ⭘ | ⭘ | ⭘ |
| 11 | Pushed the IM injection at a slow speed | ⭘ | ⭘ | ⭘ |
| 12 | Pushed the IM injection at a fast speed | ⭘ | ⭘ | ⭘ |
| 13 | Asked the patient to relax their gluteus muscle prior to injection | ⭘ | ⭘ | ⭘ |
| 14 | Administered a pain reliever prior to injection | ⭘ | ⭘ | ⭘ |
| 15 | Used topical or injectable anesthetic (i.e. lidocaine) | ⭘ | ⭘ | ⭘ |
| 17 | Other: **[PIPE THROUGH WORDING FROM Q8 (CODE 17)]** | ⭘ | ⭘ | ⭘ |

1. **Which position do you think provides optimal participant comfort with the injections?**

***Please select one answer.***

| 1 | ⭘ | Prone (lying face down) |
| --- | --- | --- |
| 2 | ⭘ | Lying on side |
| 3 | ⭘ | Standing up/bending over table/chair |
| 4 | ⭘ | Depends on participant |
| 5 | ⭘ | Position does not seem to impact patient comfort |
| 6 | ⭘ | Other |

1. **Study data on CAB + RPV LA shows that participant reporting of pain with injection decreases after the first few doses. Why do you think pain reporting declines over time?**

***Please select all that apply.***

| 1 | 🞏 | Volume decreases after 1^st^ injection (monthly dosing) |
| --- | --- | --- |
| 2 | 🞏 | Anxiety of participant decreases |
| 3 | 🞏 | Adjustment of the injection technique for the individual participant |
| 4 | 🞏 | Patients learn and post-injection pain self-management improves with time |
| 5 | 🞏 | Participant less likely to report reactions over time (study reporting bias) |
| 6 | 🞏 | Other (explain) _________ |

1. **Did you make any modifications to your injection technique for participants with larger BMI (≥30)?**

***Please select one answer.***

| 1 | ⭘ | Yes |
| --- | --- | --- |
| 2 | ⭘ | No |
| 3 | ⭘ | Not applicable. I did not inject patients with BMI ≥30 |
| 4 | ⭘ | Not applicable. I would use a longer needle (i.e. 2 inch) but unavailable in my center |

**11.1 Which modifications did you make?**

***Please select all that apply.***

| 1 | 🞏 | Used a longer (i.e. 2 inch) needle |
| --- | --- | --- |
| 2 | 🞏 | Used Z-track technique |
| 3 | 🞏 | Positioned the patient differently (please explain) _______ |
| 4 | 🞏 | Used different landmarking method to locate the injection site (please explain) _______ |

1. **Did you make any modifications to your injection technique for female participants (compared to male participants)?**

***Please select one answer.***

| 1 | ⭘ | Yes |
| --- | --- | --- |
| 2 | ⭘ | No |
| 3 | ⭘ | Not applicable. I did not inject any female participants |
| 4 | ⭘ | Not applicable. I would use a longer needle (i.e. 2 inch) but unavailable in my center |

**12.1 Which modifications did you make?**

***Please select all that apply.***

| 1 | 🞏 | Used a longer (i.e. 2 inch) needle |
| --- | --- | --- |
| 2 | 🞏 | Used Z-track technique |
| 3 | 🞏 | Positioned the patient differently (please explain) _______ |
| 4 | 🞏 | Used different landmarking method to locate the injection site (please explain) _______ |

1. **Which medication do you prefer to administer/inject first for each participant?**

***Please select one answer.***

| 1 | ⭘ | CAB first (please explain your rationale) _______ |
| --- | --- | --- |
| 2 | ⭘ | RPV first (please explain your rationale) _______ |
| 3 | ⭘ | No preference |

1. **What % of time did you use the following injection location?**

| 1 | ⭘ | Ventrogluteal injection _____ % |
| --- | --- | --- |
| 2 | ⭘ | Dorsogluteal injection (upper outer quadrant) _____ % |

**POST-INJECTION PAIN**

1. **What strategies did study participants report trying themselves to help minimize post‑injection pain or discomfort AFTER the injection?**

***Please select all that apply.***

| 1 | 🞏 | Hot packs |
| --- | --- | --- |
| 2 | 🞏 | Cold packs |
| 3 | 🞏 | Over the counter pain reliever |
| 4 | 🞏 | Resting or minimal activity |
| 5 | 🞏 | Return to routine daily activities |
| 6 | 🞏 | Stretching |
| 7 | 🞏 | Light exercise |
| 8 | 🞏 | Vigorous exercise |
| 9 | ⭘ | Participants did not report trying any strategies |
| 10 | 🞏 | Other (please specify) _________ |

**15.1 For each choice selected above, please indicate impact of strategy on pain/discomfort post-injection.**

***Please select one answer per row.***

|  |  | 1 | 2 | 3 | 4 |
| --- | --- | --- | --- | --- | --- |
|  |  | **Helped minimize post-injection** pain or discomfort of the injection | Made **post-injection** pain or discomfort of the injection **worse** | **No impact** on **post-injection** pain or discomfort of the injection | **Not sure** of the impact on **post-injection** pain or discomfort of the injection |
| 1 | Hot packs | ⭘ | ⭘ | ⭘ | ⭘ |
| 2 | Cold packs | ⭘ | ⭘ | ⭘ | ⭘ |
| 3 | Over the counter pain reliever | ⭘ | ⭘ | ⭘ | ⭘ |
| 4 | Resting or minimal activity | ⭘ | ⭘ | ⭘ | ⭘ |
| 5 | Return to routine daily activities | ⭘ | ⭘ | ⭘ | ⭘ |
| 6 | Stretching | ⭘ | ⭘ | ⭘ | ⭘ |
| 7 | Light exercise | ⭘ | ⭘ | ⭘ | ⭘ |
| 8 | Vigorous exercise | ⭘ | ⭘ | ⭘ | ⭘ |
| 10 | Other: **[PIPE THROUGH TEXT ENTERED AT Q15 (CODE 10)]** | ⭘ | ⭘ | ⭘ | ⭘ |

**15.2 Of those selected above as helpful, please rank the most effective in minimizing post‑injection pain.**

|  |  | **Most effective** in reducing post-injection pain | **Second most effective** in reducing post-injection pain | **Third most effective** in reducing post-injection pain |
| --- | --- | --- | --- | --- |
| 1 | Hot packs | ⭘ | ⭘ | ⭘ |
| 2 | Cold packs | ⭘ | ⭘ | ⭘ |
| 3 | Over the counter pain reliever | ⭘ | ⭘ | ⭘ |
| 4 | Resting or minimal activity | ⭘ | ⭘ | ⭘ |
| 5 | Return to routine daily activities | ⭘ | ⭘ | ⭘ |
| 6 | Stretching | ⭘ | ⭘ | ⭘ |
| 7 | Light exercise | ⭘ | ⭘ | ⭘ |
| 8 | Vigorous exercise | ⭘ | ⭘ | ⭘ |
| 10 | Other **[PIPE THROUGH TEXT ENTERED AT Q15 (CODE 10)]** | ⭘ | ⭘ | ⭘ |

1. **If you have any other comments or insights you would like to share around how to optimize CAB + RPV LA injections, please include below.**

Comments:

Table S1. Duration of ISR Events Through Week 96

| **Parameter** | **CAB + RPV LA dosing regimen** | | |
| --- | --- | --- | --- |
|  | **Pooled CAB + RPV LA (N=937)** | **CAB + RPV LA Q8W (n=327)** | **CAB + RPV LA Q4W  (n=610)** |
| Median duration of ISRs (IQR), days | 3 (2–4) | 3 (2–4) | 3 (2–4) |
| ISR events,^a^ n | 8453 | 2345 | 6108 |
| ISR duration 1–7 days, n (% of ISR events) | 7350 (87) | 2042 (87) | 5308 (87) |
| ISR duration >7–14 days, n (% of ISR events) | 584 (7) | 164 (7) | 420 (7) |
| ISR duration >14–30 days, n (% of ISR events) | 205 (2) | 53 (2) | 152 (2) |
| ISR duration >30 days, n (% of ISR events) | 272 (3) | 77 (3) | 195 (3) |
| ISR events reported as recovered at last  follow-up, n (% of ISR events)^b^ | 8352 (99) | 2311 (99) | 6041 (99) |

^a^Each ISR event was counted separately. A participant may have had multiple ISR events following a single injection.

^b^ISR events reported as “not recovered/resolved” may reflect the status at last follow-up and could have subsequently resolved, but not been captured. Some ISR events were reported as “recovered with sequelae” (n=59).

CAB, cabotegravir; IQR, interquartile range; ISR, injection site reaction; LA, long-acting; Q4W, every 4 weeks; Q8W, every 8 weeks; RPV, rilpivirine.

Figure S1. Participant Disposition


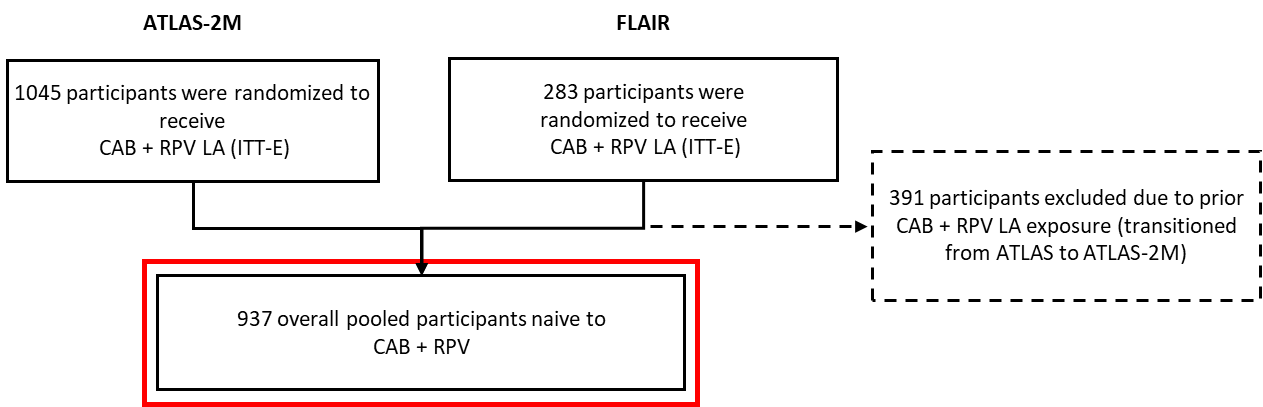


CAB, cabotegravir; ITT-E, intention-to-treat exposed; LA, long-acting; RPV, rilpivirine.

Figure S2. Duration of ISR Events Through Week 96 by ISR^a^


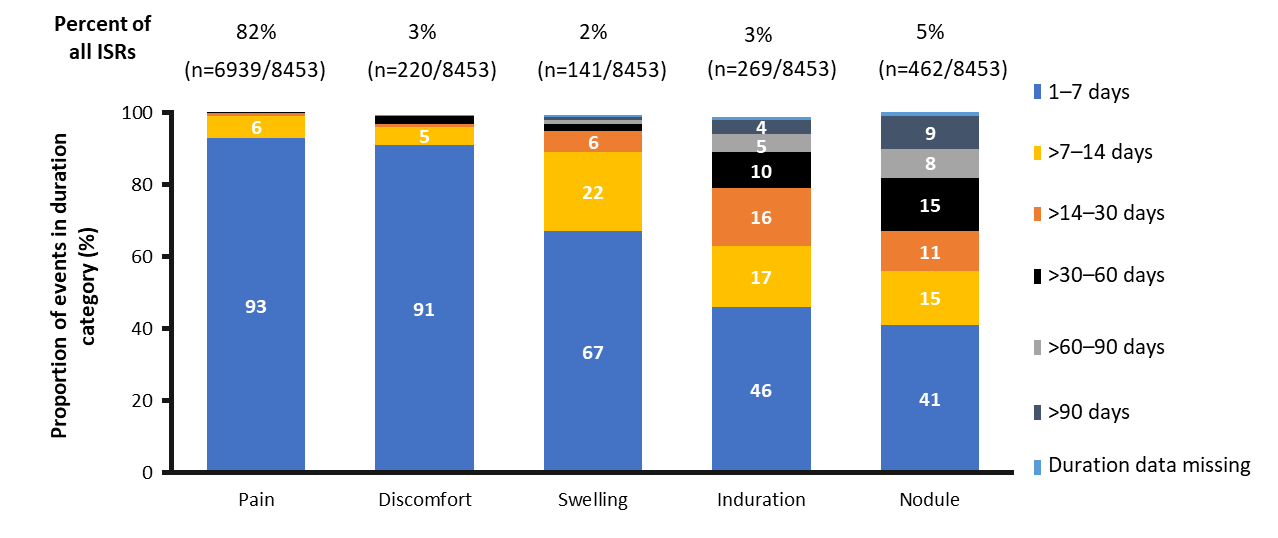
^a^Each ISR event was counted separately. A participant may have had multiple ISR events following a single injection. Top five most common ISRs reported.

ISR, injection site reaction.

Figure S3. ISRs Over Time by CAB + RPV LA Dosing Regimen Through Week 96*


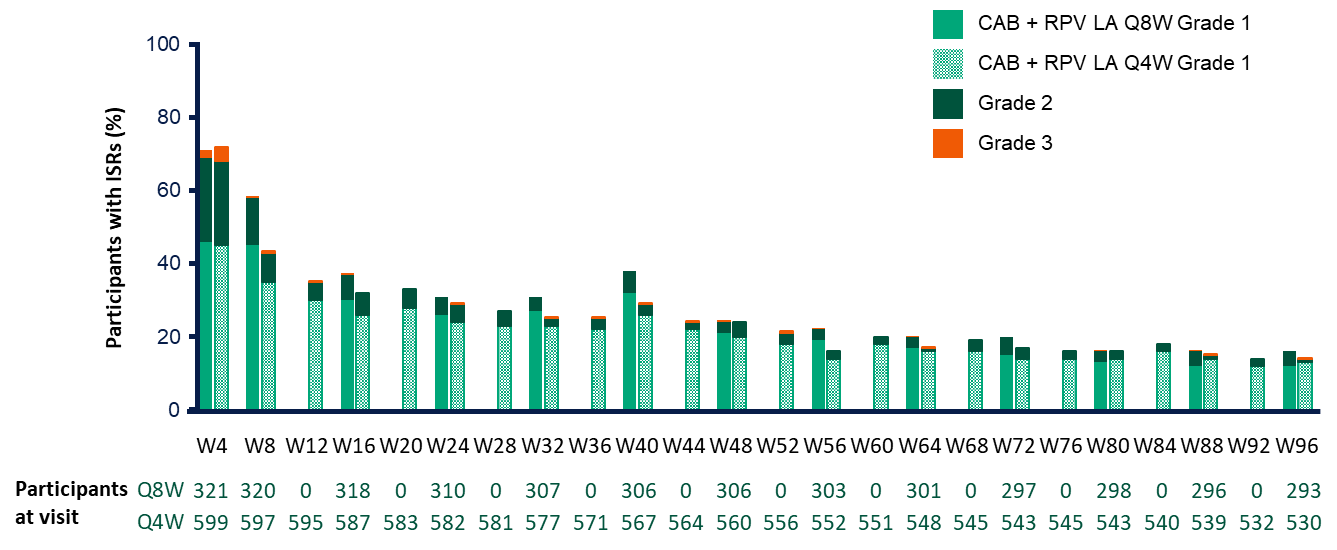
*Incidence is derived relative to the number of participants who received injections at each respective study visit. Adverse event grade is the maximum grade reported by the participant at each visit.

CAB, cabotegravir; ISR, injection site reaction; LA, long-acting; Q4W, every 4 weeks; Q8W, every 8 weeks; RPV, rilpivirine; W, week.

## Figure S4. HCP Geographic Locations


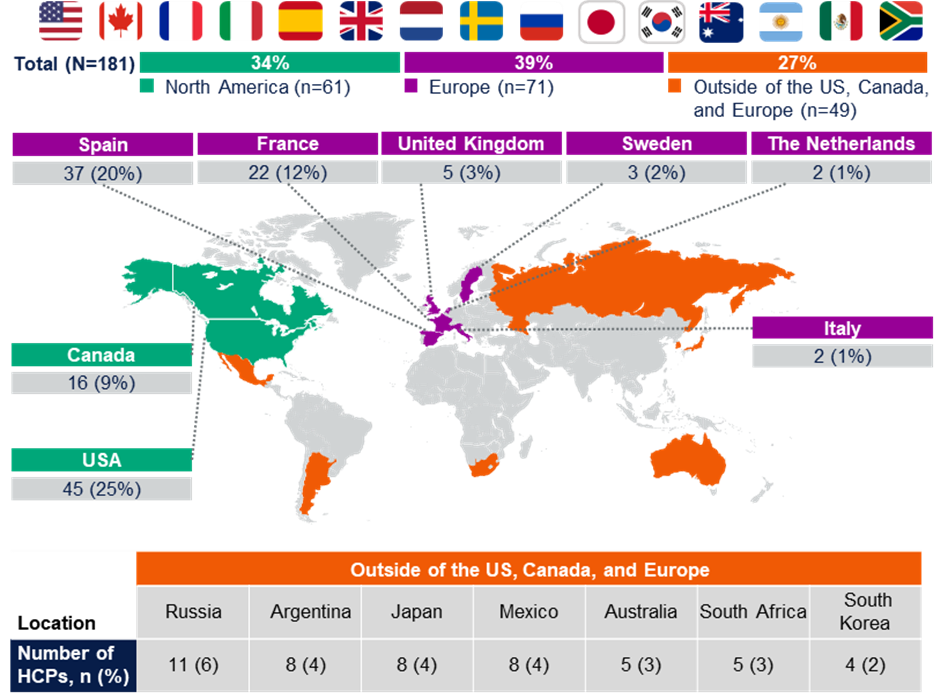


HCP, healthcare provider.

Figure S5. HCP Perceptions on Optimal Positioning for Patient Comfort
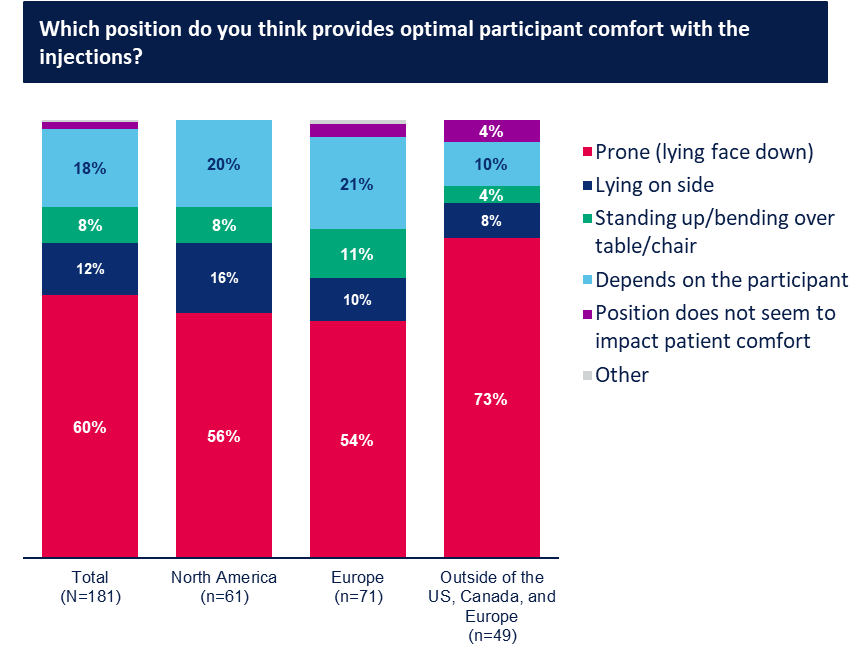


HCP, healthcare provider.

Figure S6. HCP Perceptions on Decreased Pain Reporting Over Time


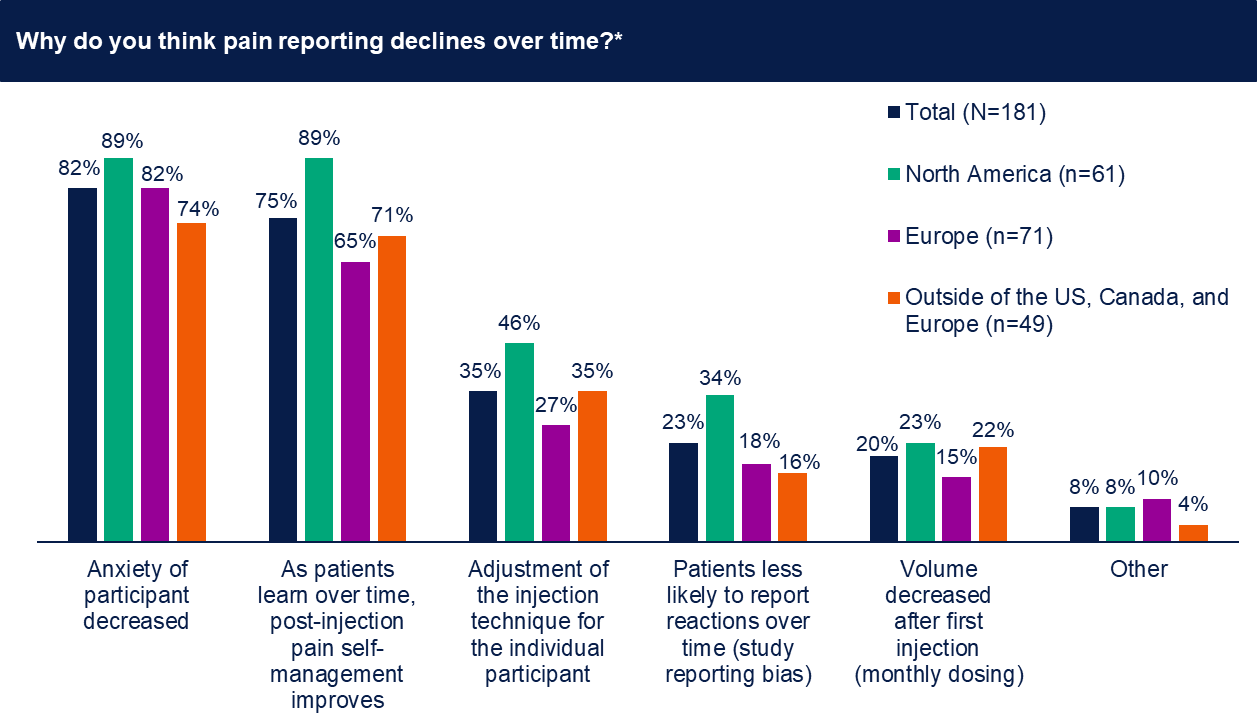
*HCPs could select more than one response to this question.

HCP, healthcare provider.

Figure S7. Injection Modifications for Participants With BMI ≥30 kg/m^2^


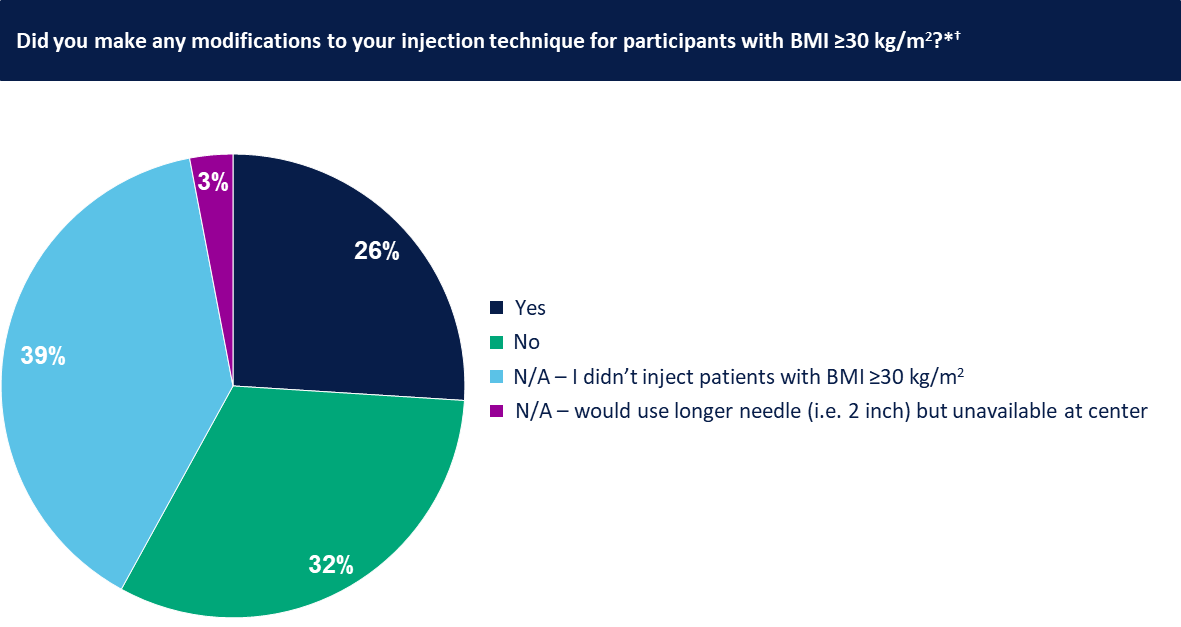


*Modifications as specified in the trial protocols. The most common injection modification was using a longer needle (i.e. 2 inches), as reported by 98% (n=46/47) of HCPs, followed by use of the Z‑track technique (23% [n=11/47]), positioning the patient differently (4% [n=2/47]), and using a different landmarking method to locate the injection site (4% [n=2/47]).

^†^Per the prescribing information, longer needle lengths may be required for patients with BMI ≥30 kg/m^2^ to ensure that injections are administered intramuscularly.^1^

BMI, body mass index; HCP, healthcare provider; N/A, not applicable.

Figure S8. Pain- and Discomfort-Minimizing Techniques Post Injection


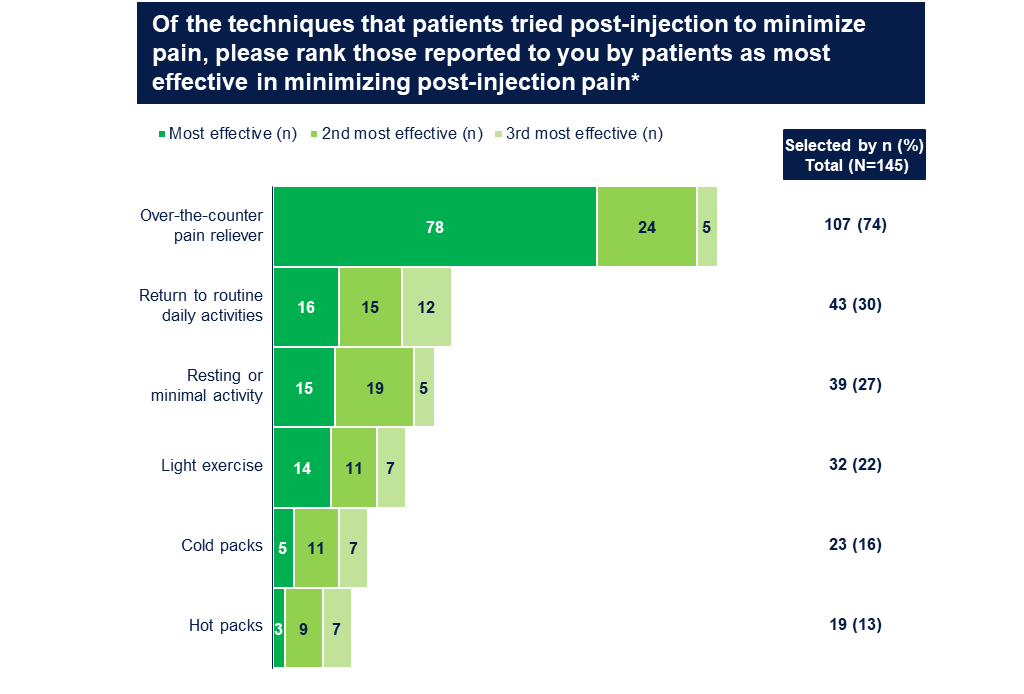
*Those reported by ≥10% of HCPs are shown. Those ranked by <10% included: vigorous exercise (8%), stretching (8%), and “other” techniques, including massaging the area and/or application of
shea butter (8%).

HCP, healthcare provider.
